# Development of a Novel Heart Failure Risk Tool: The Barcelona Bio-Heart Failure Risk Calculator (BCN Bio-HF Calculator)

**DOI:** 10.1371/journal.pone.0085466

**Published:** 2014-01-15

**Authors:** Josep Lupón, Marta de Antonio, Joan Vila, Judith Peñafiel, Amparo Galán, Elisabet Zamora, Agustín Urrutia, Antoni Bayes-Genis

**Affiliations:** 1 Heart Failure Unit, Hospital Universitari Germans Trias i Pujol, Badalona, Spain; 2 Department of Medicine, Autonomous University of Barcelona, Barcelona, Spain; 3 IMIM (Hospital del Mar Medical Research Institute), Barcelona. Spain; 4 CIBER Epidemiology and Public Health, Barcelona, Spain; 5 Biochemistry Service, Hospital Universitari Germans Trias i Pujol, Badalona, Spain; Virginia Commonwealth University, United States of America

## Abstract

**Background:**

A combination of clinical and routine laboratory data with biomarkers reflecting different pathophysiological pathways may help to refine risk stratification in heart failure (HF). A novel calculator (BCN Bio-HF calculator) incorporating N-terminal pro B-type natriuretic peptide (NT-proBNP, a marker of myocardial stretch), high-sensitivity cardiac troponin T (hs-cTnT, a marker of myocyte injury), and high-sensitivity soluble ST2 (ST2), (reflective of myocardial fibrosis and remodeling) was developed.

**Methods:**

Model performance was evaluated using discrimination, calibration, and reclassification tools for 1-, 2-, and 3-year mortality. Ten-fold cross-validation with 1000 bootstrapping was used.

**Results:**

The BCN Bio-HF calculator was derived from 864 consecutive outpatients (72% men) with mean age 68.2±12 years (73%/27% New York Heart Association (NYHA) class I-II/III-IV, LVEF 36%, ischemic etiology 52.2%) and followed for a median of 3.4 years (305 deaths). After an initial evaluation of 23 variables, eight independent models were developed. The variables included in these models were age, sex, NYHA functional class, left ventricular ejection fraction, serum sodium, estimated glomerular filtration rate, hemoglobin, loop diuretic dose, β-blocker, Angiotensin converting enzyme inhibitor/Angiotensin-2 receptor blocker and statin treatments, and hs-cTnT, ST2, and NT-proBNP levels. The calculator may run with the availability of none, one, two, or the three biomarkers. The calculated risk of death was significantly changed by additive biomarker data. The average C-statistic in cross-validation analysis was 0.79.

**Conclusions:**

A new HF risk-calculator that incorporates available biomarkers reflecting different pathophysiological pathways better allowed individual prediction of death at 1, 2, and 3 years.

## Introduction

Risk stratification of heart failure (HF) is a challenge, and as guidelines acknowledge, new accurate scoring models are needed. Several models have been developed [1]–[10], of which the Seattle HF model [6] has had the most visibility. Nevertheless, this scoring model is derived from a cohort of patients carefully selected for a randomized clinical trial over 20 years ago, with its inclusion and exclusion criteria. Serum biomarkers for patient risk stratification were not available. However, in recent times a number of biomarkers reflective of different pathophysiological pathways have been identified in HF [11]. Therefore, we developed a calculator for HF stratification that, in addition to classical risk factors, includes N-terminal pro B-type natriuretic peptide (NT-proBNP), a marker of myocardial stretch; high-sensitivity cardiac troponin T (hs-cTnT), a marker of myocyte injury; and high-sensitivity soluble ST2 (ST2), which is reflective of myocardial fibrosis and remodeling.

The Barcelona Bio Heart Failure risk calculator (BCN Bio-HF calculator), which is derived from a real-life cohort of contemporary treated HF patients, is a web-based calculator allowing quick and easy interactive calculations of individual mortality at 1, 2, and 3 years and life expectancy.

## Methods

### Derivation Study Population

The study population, samples, and biomarker assays were described elsewhere [12]. In summary ambulatory patients treated at a multidisciplinary HF unit were consecutively included in the study in an outpatient setting. Patients were referred to the unit by cardiology or internal medicine departments and, to a lesser extent, from the emergency or other hospital departments. The principal referral criterion was HF according to the European Society of Cardiology guidelines irrespective of etiology, at least one HF hospitalization, and/or reduced LVEF. Etiologies of HF were: ischemic heart disease 52.2%, dilated cardiomyopathy 10%, hypertensive 9.4%, alcoholic cardiomyopathy 5.7%, drug-related cardiomyopathy 2.5%, valvular disease 11.4% and others 8.8%.

All participants provided written informed consent, and the local ethics committee approved the study. All study procedures were in accordance with the ethical standards outlined in the Helsinki Declaration of 1975 as revised in 1983. The regular visitation schedule was reported elsewhere [12]–[15]. Death from all causes was the main outcome. Fatal events were identified from clinical records, family contact or by reviewing the electronic clinical history at the Catalan and Spanish Institute of Health. Physicians and nurses of the HF Unit identified adverse events (JL, M de A, AU, BG, RC, LC).

### Model Making

A selection of 23 well-known mortality-related variables from the literature and from previous own studies was first evaluated, and 11 of them were included in eight Cox proportional hazard regression models due to their significance in the multivariate analysis or because considered of clinical significant relevance: one model without biomarkers (‘clinical model’) and seven additional models with all possible combinations of the three biomarkers.

Proportional assumptions needed to use Cox proportional hazard regression models were tested for all variables. Variables in which the non-linear component achieved significance were transformed according to what the figure of time vs. hazard suggested until non-significance of the non-linear component was achieved, as reported elsewhere [12], [14], [15]. In summary, to fulfill the assumption of linearity for the co-variables hs-cTnT, ST2, and NT-proBNP, the logarithmic functions of both NT-proBNP and hs-cTnT, the quadratic term of the logarithmic function of hs-cTnT, and the quadratic term of ST2 were used in the Cox proportional hazard regression models. In the ‘clinical model’, variables were removed one-by-one in a backward manner to assess whether their exclusion significantly reduced the likelihood of the model. When two variables were collinear in predicting outcome, the one with the better likelihood was included. All two-variable interactions were also tested. Some variables were dichotomized (such as New York Heart Association (NYHA) functional class or left ventricular ejection fraction (LVEF) for better performance).

### Model Performance

We used different measures of performance to test the potential incremental prognostic value of the three biomarkers as follows:

#### Discrimination

The ability of the model to discriminate between patients who will have and will not have the event along all follow-up was measured by means of the C-statistic obtained from a generalization of Somers ‘Dxy’ rank correlation, which equals 2×(c−1/2), where c is the concordance (discrimination) probability [16], which already incorporates information from censored data.

#### Calibration

How well the observed incidence rate fit the predicted risk was measured by Nam-D'Agostino statistics using the Hosmer and Lemeshow test for censored survival [17]. Calibration using this method was calculated for one-, two- and three-year mortality.

#### Accuracy

The integrated Brier score for censored observations was used to measure the accuracy of probabilistic predictions [18]. A lower score represents higher accuracy. This score takes values between 0 and 1 and was calculated for one-, two- and three-year mortality.

#### Best prediction

The Bayesian information criterion (BIC) and the Akaike information criterion (AIC), measures of the relative goodness-of-fit of a statistical model, were used to compare non-nested models. Lower values indicate a better model along all follow-up. Both indicators take into account the events along all follow-up.

#### Reclassification

We used the method described by Pencina et al. [19]. Integrated discrimination improvement (IDI) considers changes in the estimated mortality prediction probabilities as a continuous variable. Net reclassification improvement (NRI) requires a previous definition of meaningful risk categories; we used tertiles for the risk of death: <18.5%, 18.5–41%, and >41%. NRI considers changes in the predicted probabilities of estimated mortality that imply a change from one category to another. Reclassification was evaluated for one-, two- and three-year mortality.

#### Generalization or validation

To assess how the results of the models can be generalized to an independent data set, a 10-fold cross-validation technique was used [20]. Using a bootstrapping technique, we created 1000 samples (allowing repetition) equal in size to the present cohort. One by one, each of the 1000 samples was split into 10 distinct blocks roughly equal in size. We left out the first block (the testing set) and fit a model with the remaining blocks (the training set) to predict the held-out-block. We continued this process until the model predicted all 10 held-out-blocks. The mean C-statistic was calculated and the process repeated for all 1000 samples.

### Calculator algorithms

#### Mortality

The calculator was designed to run with the availability of none, one, two, or three of the chosen biomarkers, using the best model for each available combination. To calculate the probability of developing an event at a specific time, the following formula was applied:
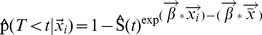



Where:

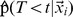
  =  probability to develop the event at time “T” before time “t” (e.g., 3 years) giving a combination “
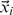
” of patient characteristics (i.e., age, sex, etc.);

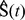
  =  estimated survival at time “t”;

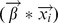
  =  the vector of beta coefficients (natural log of the hazard ratio (HR)) multiplied by the vector of patient characteristics;

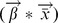
  =  the vector of beta coefficients multiplied by the vector of mean covariates;

#### Life expectancy

To get an estimate of life expectancy we refitted all Proportional Hazard Cox-Regression models in parametric Weibull models [21]. In those models the mean survival, “E(*T*)”, is estimated by:
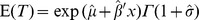
where,


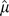
 is the estimated intercept obtained from the model


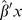
 is the product of the coefficients and patient's characteristics


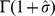
 is call the gamma function of the 1 plus the estimated “scale” parameter obtained from the model.

Statistical analyses were performed using R software version 2.15.2. (http://www.R-project.org/).

## Results

After an initial evaluation of 23 variables, eight models (one without biomarkers and seven with combinations of the three studied biomarkers) were finally included in the risk calculator tool.

Table 1 provides the evaluated demographic, clinical, and biochemical characteristics of the studied patients with univariate and multivariate Cox regression analysis. During a median follow-up of 3.4 years (25^th^-75^th^ percentiles 1.8–5.0 years) 305 deaths occurred. The follow-up for alive patients was 4.4 years (25^th^–75^th^ percentiles 2.7–5.2). The following variables emerged as significant in at least one of the models: age, sex, NYHA functional class, LVEF, estimated glomerular filtration rate (eGFR), serum sodium, hemoglobin, daily loop diuretic dose, beta-blocker, angiotensin converting enzyme inhibitor (ACEI)/angiotensin-2 receptor blocker (ARB), statin treatment and hs-cTnT, ST2, and NTproBNP levels. No pair-wise interaction between variables achieved significance. Variables excluded from the models due to the lack of statistical improvement in the model were: ischemic etiology of HF, diabetes mellitus, body mass index, blood systolic pressure, heart rate, atrial fibrillation, chronic obstructive pulmonary disease, hypertension, iron deficiency, cystatin-C, spironolactone/eplerenone treatment, cardiac resynchronization therapy (CRT), and implantable cardiac defibrillator (ICD).

**Table 1:** Demographic, clinical and analytical characteristics of patients.

	NO EVENT	EVENT	Univariate analysis		Multivariate analysis without biomarkers		Multivariate analysis with biomarkers	
	N = 559	N = 305						
			HR_Cox_	P-value	HR_Cox_	P-value	HR_Cox_	P-value
Age, years	64.6 (12.3)	74.4 (9.23)	1.07 [1.06;1.08]	<0.001	1.04 [1.03;1.06]	<0.001	1.04 [1.02;1.06]	<0.001
Female Gender	149 (26.7%)	93 (30.5%)	1.07 [0.84;1.36]	0.590	0.66 [0.5;0.89]	0.005	0.77 [0.57;1.03]	0.078
BMI, Kg/m^2^	28.0 (4.95)	27.0 (5.15)	0.96 [0.93;0.98]	<0.001	1.00 [0.97;1.02]	0.724	1.01 [0.98;1.04]	0.695
NYHA III–IV	91 (16.3)	144 (47.2)	3.25 [2.59;4.07]	<0.001	1.87 [1.45;2.42]	<0.001	1.67 [1.29;2.17]	<0.001
LVEF,%	35.6 (13.0)	36.5 (15.0)	1.00 [0.99;1.01]	0.801				
LVEF >45%	122 (21.8%)	76 (24.9%)	0.98 [0.75;1.28]	0.905	0.76 [0.54;1.06]	0.108	0.95 [0.67;1.34]	0.770
Ischemic etiology of HF	286 (51.2%)	165 (54.1%)	1.06 [0.84;1.32]	0.633	1.22 [0.92;1.6]	0.162	1.14 [0.86;1.5]	0.365
Systolic Blood Pressure, mmHg	127 (23.1)	127 (22.9)	1.00 [0.99;1.00]	0.775	1.00 [0.99;1.00]	0.605	1.00 [0.99;1.01]	0.872
Heart rate, bpm	68.9 (13.5)	70.7 (13.7)	1.01 [1.00;1.02]	0.004	1.00 [0.99;1.01]	0.469	0.99 [0.9;1]	0.115
Diabetes mellitus	181 (32.4%)	129 (42.3%)	1.42 [1.14;1.79]	0.002	1.26 [0.98;1.63]	0.070	1.2 [0.93;1.55]	0.166
Atrial fibrillation	82 (14.7%)	63 (20.7%)	1.49 [1.13;1.97]	0.004	1.09 [0.8;1.48]	0.583	0.96 [0.7;1.32]	0.796
Hypertension	327 (58.5%)	200 (65.6%)	1.31 [1.04;1.66]	0.024	0.97 [0.74;1.27]	0.836	0.89 [0.68;1.16]	0.388
Sodium, mmol/L	139 (3.22)	139 (3.79)	0.94 [0.90;0.97]	<0.001	0.94 [0.91;0.97]	<0.001	0.96 [0.93;1]	0.03
COPD	71 (12.7%)	73/23.9%)	1.69 [1.30;2.20]	<0.001	1.11 [0.83;1.5]	0.480	1.08 [0.79;1.47]	0.633
eGFR, ml/min/1.73m2	52.0 (24.2)	35.8 (16.7)	0.97 [0.96;0.97]	<0.001	0.99 [0.98;1]	0.019	1 [0.99;1.01]	0.800
Hemoglobin, g/dl	13.3 (1.72)	12.3 (1.81)	0.77 [0.72;0.82]	<0.001	0.90 [0.84;0.97]	0.008	0.92 [0.85;0.99]	0.035
Iron defficiency	260 (46.8%)	174 (57.2%)	1.42 [1.13;1.79]	0.002	1.06 [0.83;1.36]	0.627	0.98 [0.76;1.26]	0.847
ACEI or ARB	523 (93.6%)	251 (82.6%)	0.35 [0.26;0.47]	<0.001	0.69 [0.49;0.95]	0.024	0.91 [0.63;1.31]	0.601
Beta-Blocker	523 (93.6%)	233 (76.4%)	0.35 [0.27;0.45]	<0.001	0.58 [0.42;0.79]	<0.001	0.61 [0.44;0.84]	0.003
Statins	420 (75.1%)	172 (56.4%)	0.50 [0.40;0.63]	<0.001	0.57 [0.43;0.74]	<0.001	0.61 [0.46;0.81]	<0.001
Loop diuretic dose:								
0 (no loop diuretic)	107 (19.1%)	26 (8.52%)	Ref.	Ref.	Ref.	Ref.	Ref.	Ref.
Dose 1†	361 (64.6%)	172 (56.4%)	1.81 [1.20;2.73]	0.005	1.27 [0.81;2]	0.291	1.26 [0.69;1.16]	0.384
Dose 2‡	91 (16.3%)	107 (35.1%)	3.73 [2.43;5.73]	<0.001	1.75 [1.07;2.97]	0.025	1.5 [0.9;2.48]	0.119
Spironolactone/eplerenone	225 (40.3%)	115 (37.7%)	1.03 [0.82;1.30]	0.780	1.02 [0.79;1.32]	0.864	0.89 [0.82;1.30]	0.780
CRT	31 (5.55%)	16 (5.25%)	0.84 [0.50;1.38]	0.483	0.92 [0.53;1.59]	0.761	0.85 [0.49;1.47]	0.558
ICD	66 (11.8%)	26 (8.52%)	0.69 [0.46;1.04]	0.074	1 [0.64;1.55]	0.988	0.96 [0.62;1.5]	0.867
Cystatin-C, mg/L	1.09 [0.90;1.39]	1.48 [1.15;1.93]	2.69 [2.21;3.28]	<0.001			1.15 [0.68;1.96]	0.602
Hs-cTnT, ng/L	15.7 [7.90;30.7]	34.1 [20.5;53.6]	11.6 [5.46;24.8]	<0.001			3.79 [1.62;8.85]	0.002
ST2, ng/mL	35.5 [29.4;45.5]	44.7 [33.9;60.0]	1.48 [1.34;1.65]	<0.001			1.23 [1.09;1.38]	<0.001
NTproBNP, ng/L	975 [361;2376]	2215 [935;5193]	1.61 [1.48;1.75]	<0.001			1.13 [0.99;1.29]	0.073

Data are expressed as mean (standard deviation), median [percentiles 25^th^–75^th^] or absolute number (percentage).

ACEI =  angiotensin-converting enzyme inhibitor; ARB =  angiotensin II receptor blocker; BMI =  body mass index; COPD: Chronic obstructive pulmonary disease; eGFR =  estimated glomerular filtration rate; HF, heart failure; hs-cTnT, high-sensitivity circulating troponin T; ST2 =  high-sensitivity soluble ST2; LVEF =  left ventricular ejection fraction; NT-proBNP =  N-terminal pro-brain natriuretic peptide; NYHA =  New York Heart Association. CRT =  cardiac resynchronization therapy; ICD =  implantable cardiac defibrillator.

Ref.  =  Reference.

Loop diuretic dose 1: Furosemide-equivalent dose up to 40 mg/day or Torasemide up to 10 mg/day.

Loop diuretic dose 2: Furosemide-equivalent dose >40 mg/day or Torasemide>10 mg/day.

The logarithmic functions of NTproBNP, hs-cTnT and cystatin C, the quadratic term of the logarithmic function of hs-cTnT, ST2 as ST2/10 and the quadratic term of ST2/10 were used in the Cox models. P value for (ST2/10)^2^<0.001 in the univariate analysis and 0.006 in the multivariate analysis; P value for log(hs-cTnT)^2^<0.001 in the univariate analysis and 0.015 in the multivariate analysis.

In the ‘clinical model’ (Model 1), LVEF (HR 0.69, P = 0.016), eGFR (HR 0.99, P = 0.017), and ACEI/ARB treatment (HR 0.67, P = 0.014) were significant outcome predictors. In the other models, these variables often lost significance after the addition of biomarkers. In Model 8, in which the predictors were adjusted for the three biomarkers, age (HR 1, P<0.001) and NYHA functional class (HR 1.67, P<0.001) remained strong risk factors, whereas female sex (HR 0.75, P = 0.029), statin treatment (HR 0.67, P = 0.001), serum sodium (HR 0.96, P = 0.036), plasma hemoglobin (HR 0.91, P = 0.006), and beta-blocker treatment (HR 0.60, P<0.001) showed a significant protective effect. The three biomarkers exhibited a relationship with mortality, but in model 8, NTproBNP only showed a prognostic trend (Log(hs-TnT): HR 3.38, P = 0.002; ST2/10: HR 1.23, P<0.001; and Log(NTproBNP): HR 1.11, P = 0.078).

**Figure 1:**
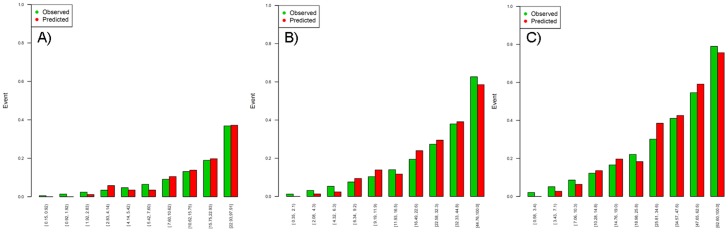
Observed and predicted mortality according to risk deciles (Hosmer and Lemeshow test) at 1-year (A), 2-year (B), and 3-year (C) follow-up for model 8 (with the three biomarkers).

To calculate the probabilities to develop an event at specific time for a particular covariates combination, beta coefficients, survival at the mean of covariates and the sum of the product of coefficients per covariates mean are needed. Survival at the mean of covariates was 94.2% at 1 year, 87.5% at 2 years and 80.2% at 3 years. The remaining values are shown in Table S1 in File S1. When a covariate added no increased prognostic accuracy, it was not included in the risk calculation. An example of calculator functioning is shown in the appendix. Table 2 shows the C-statistic for the ‘clinical model’ and all of the models containing biomarkers (alone or in combination) in the derivation sample. The model with the three biomarkers had a C-statistic of 0.794 (95% CI 0.77;0.817). Calibration for 1-, 2-, and 3-year mortality was good (non-significant in the Hosmer and Lemeshow test) (Fig. 1).

**Table 2:** Performance of the models.

	Clinical	Clinical model	Clinical model	Clinical model	Clinical model	Clinical model	Clinical model	Clinical model
	Model	+ NTproBNP	+ hs-cTnT	+ ST2	+ NTproBNP	+ NTproBNP	+ hs-cTnT	+ all biomarkers
					+ ST2	+ hs-cTnT	+ ST2	
**C-statistic**	0.771	0. 777	0.782	0.779	0.785	0.784	0.792	0.793
	(0.744;0.797)	(0.751;0.803)	(0.758;0.807)	(0.754;0.804)	(0.76;0.809)	(0.759 0.809)	(0.768;0.815)	(0.77;0.817)
	Reference	P-value = 0.052	P-value = 0.061	P-value = 0.098	P-value = 0.015	P-value = 0.021	P-value = 0.003	P-value = 0.001
**H-L**								
1 year	?^2^: 8.3	?^2^: 4.23	?^2^: 5.54	?^2^: 5.34	?^2^: 7.51	?^2^: 4.46	?^2^: 10.6	?^2^: 8.67
	P-value = 0.50	P-value = 0.90	P-value = 0.79	P-value = 0.80	P-value = 0.58	P-value = 0.81	P-value = 0.23	P-value = 0.37
2 years	?^2^: 10.6	?^2^: 6.52	?^2^: 5.82	?^2^:10.03	?^2^:10.89	?^2^: 7.41	?^2^:8.03	?^2^:7.46
	P-value = 0.31	P-value = 0.69	P-value = 0.76	P-value = 0.35	P-value = 0.28	P-value = 0.49	P-value = 0.43	P-value = 0.49
3 years	?^2^:10.4	?^2^: 5.16	?^2^:7.63	?^2^:14.87	?^2^:11.20	?^2^:11.15	?^2^:7.53	?^2^:7.78
	P-value:0.32	P-value:0.82	P-value:0.57	P-value:0.094	P-value:0.26	P-value:0.19	P-value:0.48	P-value:0.46
**Brier score**								
1 year	0.252	0.251	0.250	0.250	0.249	0.249	0.247	0.247
2 years	0.198	0.195	0.193	0.193	0.192	0.191	0.188	0.188
3 years	0.169	0.166	0.163	0.164	0.161	0.161	0.157	0.157
**AIC**	3470	3455	3440	3455	3441	3435	3421	3421
**BIC**	3527	3517	3488	3512	3498	3492	3473	3478
**IDI**								
1 year		−0.02 [−0.62;0.57]	0.45 [−0.18; 1.08]	0.57 [−0.12;1.26]	0.54 [−0.20;1.28]	0.59 [−0.03; 1.20]	0.97 [0.15;1.80]	0.92 [0.11;1.72]
	Reference	P-value = 0.94	P-value = 0.16	P-value = 0.11	P-value = 0.15	P-value = 0.063	P-value = 0.021	P-value = 0.025
2 years		−0.02 [−0.92;0.88]	1.03 [0.03; 2.04]	0.79 [−0.22; 1.79]	0.85 [−0.27; 1.97]	1.32 [0.32; 2.31]	1.84 [0.56; 3.11]	1.80 [0.56; 3.04]
	Reference	P-value = 0.972	P-value = 0.044	P-value = 0.126	P-value = 0.136	P-value = 0.009	P-value = 0.005	P-value = 0.005
3 years		0.07 [−1.01;1.16],	1.52 [0.27; 2.78]	0.86 [−0.32; 2.04]	1.12 [−0.22; 2.45]	1.95 [0.70; 3.21]	2.55 [0.99; 4.10]	2.57 [1.05; 4.09]
	Reference	P-value = 0.89	P-value = 0.017	P-value = 0.15	P-value = 0.10	P-value = 0.002	P-value = 0.001	P-value = <0.001
**NRI**								
1 year		2.34 [−4.29;8.97]	7.03 [−0.77;14.84]	3.96 [−2.55;10.47]	11.36[4.09;18.63]	8.47 [0.95;15.99]	10.34[1.92;18.76]	9.58 [1.49;17.66]
	Reference	P-value = 0.489	P-value = 0.077	P-value = 0.233	P-value = 0.002	P-value = 0.027	P-value = 0.016	P-value = 0.02
2 years		2.35 [−4.24;8.95]	6.83 [−1.00;14.66]	3.96 [−2.55;10.47]	10.63[3.27;17.98]	7.96 [0.33;15.59]	10.09[1.64;18.53]	8.80 [0.77;16.82]
	Reference	P-value = 0.48	P-value = 0.087	P-value = 0.23	P-value = 0.005	P-value = 0.041	P-value = 0.019	P-value = 0.032
3 years		2.15 [−4.42;8.73]	7.09 [−0.80;14.97]	3.74 [−2.80;10.28]	10.92[3.63;18.21]	7.96 [0.35;15.57]	9.76 [1.23;18.28]	8.93 [0.95;16.92]
	Reference	P-value = 0.52	P-value = 0.078	P-value = 0.26	P-value = 0.003	P-value = 0.04	P-value = 0.025	P-value = 0.028

χ^2^ = Chi-Square.

P values vs Model 1 (Clinical).

AIC =  Akaike information criterion; BIC =  Bayesian information criterion; HL =  Hosmer–Lemeshow test; hs-cTnT, =  high-sensitivity circulating troponin T;

ST2 =  high-sensitivity soluble ST2; IDI =  integrated discrimination improvement; NRI =  net reclassification improvement. NT-proBNP =  N-terminal pro-brain natriuretic peptide.

Model 1 (Clinical)  =  Age, Female, NYHA functional class (as I−II vs III−IV), LVEF (as ≥ vs <45%), eGFR (estimated glomerular filtration rate), Sodium,

Hemoglobin, Furosemide-equivalent doses treatment as 0, ≤40 mg/day and >40 mg/day or Torasemide as 0, ≤10 mg/day and >10 mg/day),

ACEI or ARB treatment, beta-blocker treatment, statin treatment.

Model 2 =  Model 1+NT-proBNP.

Model 3 =  Model 1+hs-cTnT.

Model 4 =  Model 1+ST2.

Model 5 =  Model 1+NT-proBNP+ST2.

Model 6 =  Model 1+NT-proBNP+hs-cTnT.

Model 7 =  Model 1+hs-cTNT+ST2.

Model 8 =  Model 1+NT-proBNP+hs-cTNT.

Reclassification for 1-, 2-, and 3-year mortality was better in the models containing more than one biomarker, with the highest found using the combination of ST2 and hs-cTnT (Model 7; Table 2). The best overall performance was observed with models 7 and 8 (Table 2).

**Figure 2:**
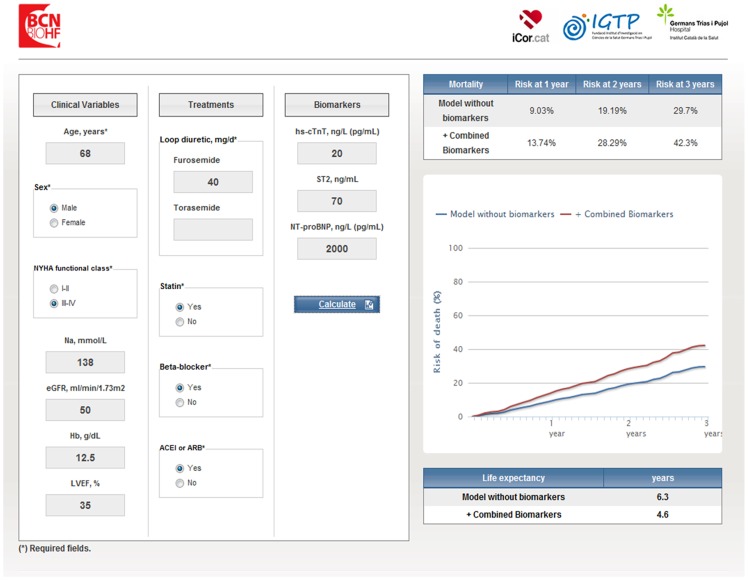
The Barcelona Bio-HF calculator has been implemented as an interactive program that employs the eight developed models to estimate 1-, 2-, and 3-year mortality for an individual patient. This tool is available at www.BCNBioHFcalculator.cat.

A web-based calculator (Fig. 2) (www.BCNBioHFcalculator.cat) has been developed, allowing interactive calculation of estimated individual probability. A graphic with monthly mortality probabilities is also available. Risk of death was found to be largely influenced by biomarkers' results. As a practical exemple a 68 year-old male in NYHA class III, LVEF 30%, sodium 130 mmol/L, eGFR 45 ml/min/m2, hemoglobin 12 g/dl, taking 60 mg of furosemide and on treatment with statins, ACEI and betablockers had a risk of death of 22%, 42% and 60% at 1,2 and 3 years, respectively. When adding the following biomarker levels: hs-cTnT 14 ng/L, ST2 40 ng/mL and NTproBNP 900 ng/L, the risk fell to 10%, 21% and 32%. However, if biomarker data had been hs-cTnT 70 ng/L, ST2 140 ng/mL, and NTproBNP 2500 ng/L the risk would rise to 35%, 62% and 80%, respectively (Fig. S1).

**Figure 3:**
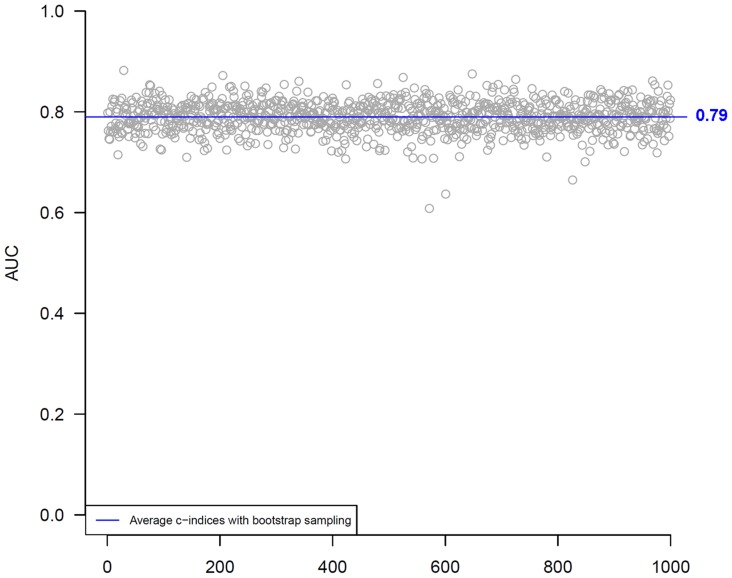
Ten-fold cross-validation with 1000 bootstrapping.

In the 10-fold cross-validation analysis with 1000 bootstrapping, the average C-statistic for the model with all combined biomarkers was 0.79 (Fig. 3), suggesting that the results may be generalized safely to independent data sets.

Mean (95% confidence interval) life expectancy for the entire cohort was 11.8 years (11.1−12.4) and expected mean age (95% confidence interval) for death was 80.2 years (79.7−80.7) using clinical model; and 11.4 years (10.7−12.0) and 79.8 years (79.3−80.3) with Model 8 (containing the three biomarkers), respectively. Beta-coefficients of the different Weibull models used to calculate life expectancy are shown in Table S2 in File S1.

## Discussion

HF risk prediction is a cornerstone of HF management. The development of an accurate HF risk calculator has the potential for tailored management. The BCN Bio-HF calculator reported here was derived from a real-life contemporarily treated consecutive cohort and includes, in addition to conventional prediction factors, three serum biomarkers (NTproBNP, ST2, and hs-cTnT) that are highly accurate for cardiac malfunction.

Mortality risk prediction models specific to the HF population have been developed with broad variation in the degree of validation and concretion of prognostic output, from classification into risk groups (low-high risk, low-medium-high risk, risk deciles) [2]–[5], to life expectancy [6] or individual mortality at a certain time point [3], [5−7], . Most of these models have not included a substantial proportion of patients taking evidence-based treatments, including ACEI/ARBs, beta-blockers, and spironolactone/eplerenone, or were developed only for patients admitted to the hospital [8], [9]. In the Seattle HF Model, the relative effect for HF medications could not be obtained from the derivation cohort and benefits were estimated from published trials or meta-analyses. In our cohort of ambulatory patients, 87% were on beta-blockers, 90% on ACEI/ARBs, and 40% on spironolactone/eplerenone. Independent of the causality of the risks and benefits of treatments, taking evidence-based HF drugs clearly reduces the risk of death, and they merit inclusion in a risk calculator. In fact, the estimated risk can be very significantly modified by treatment, both in the model without biomarkers and in the model containing biomarkers where treatment can also modify their level and the calculated risk of death would consequently change.

Some scores [4]–[6] have the advantage of a large derivation cohort. Their limitation is that all of the subjects in their derivation and validation samples were participating in a clinical trial and how well they represent those in routine clinical practice is unknown. In addition, all of the samples were obtained over a decade ago, and none included biomarker testing. The very recent 3C-HF score [7] did include contemporary treatment but not biomarkers in a mixed population of in- and outpatients included both prospectively and retrospectively. Also the even more recent MAGGIC score, derived from a metaanalysis of 30 studies [10] did not include biomarkers. In the discussion, however, the authors of this score state: “Any new risk score's success depends on the patient variables available for inclusion. Current knowledge of biomarkers in HF is inevitably ahead of what data are available across multiple cohort studies… but could not be included in our model. In principle, its inclusion would enhance further the excellent prognostic discrimination we achieved with routinely collected longestablished predictors.” The inclusion of B-type natriuretic peptide in the Valsartan-Heart Failure Trial to the Seattle HF Model increased AUC by ≈ 0.03 [6]. In the BCN Bio-HF Calculator, we included three commercially available complementary biomarkers that provide information about myocyte necrosis (hs-cTnT), fibrosis, remodeling, and inflammation (ST2), and chamber strain (NTproBNP). Other biomarkers are in the pipeline for the HF field, but some of them are not yet commercially available (i.e. growth differential factor-15) and others reflect pathways that overlap those used here. We and others previously reported on the prognostic utility of these three biomarkers [12], [14], [15]. This calculator was developed with eight models that include none, one, two, or the three biomarkers, allowing its use with any combination of biomarkers. This characteristic is unique to this new tool, which in combination with the use of state-of-the-art statistics for biomarker values, which include C-statistic, as well as calibration and reclassification, makes it more robust.

The Seattle HF Model [6] is probably the most extensively used model. It was prospectively validated in several trials. The validation AUC varied from 0.68 to 0.81 in these diverse populations, with an overall AUC of 0.73 and an AUC of less than 0.70 in the three biggest cohorts [6]. AUC from other studies rank from 0.75 (CHARM two-year mortality [4]) to 0.83 in the validation cohort of the 3C-HF score (one-year mortality with logistic regression analysis [5]). The use of Somers ‘Dxy’ rank correlation in the C-statistic analysis, which already incorporates information from censored data, is more correct from the survival point of view rather than determination of C-statistic for death at one fixed point with logistic regression models. The C-statistic analysis using logistic regression model in our population was 0.82 for 1-year, 0.82 for 2-year and 0.83 for 3-year mortality. We evaluated both the Seattle HF Model and the 3C-HF score in our population. Taking into account the inherent limitations (default values of Seattle HF for percentage of lymphocytes as well as “diabetes” instead of “diabetes with organ damage” for 3C-HF) the C-statistic using Somers ‘Dxy’ rank correlation in such models were 0.71 (95% CI 0.678-0.79) for the Seattle HF model and 0.73 (95% CI 0.68-0.73) for the 3C-HF score.

The validation obtained in our 10-fold cross-validation analysis with 1000 bootstrapping was substantially higher, averaging 0.79. Cross-validation is useful, especially when additional samples are hazardous, costly, or impossible to collect. The resulting average accuracy is likely somewhat of an underestimate for the true accuracy when the model is trained on all data and tested on external data (the optimal way for validation), but in most cases this estimate is reliable, particularly if the amount of available data is sufficiently large and the external data follows the same distribution as the available data [22]. Both the Seattle HF Model and the BCN Bio-HF calculator provide the individual risk of death at several points of time without the necessity of a physician calculating the score as an intermediate step. Also, as an added value to other scores, both allow predicting life expectancy, although using different statistical methods.

A number of the clinical variables in our calculator are also included in the Seattle HF Model, though the former has fewer variables. Some variables that may be considered clinically important, such as devices, aldosterone blockers, and systolic blood pressure, were excluded from our model due to the absence of significance in the multivariable model. In the case of devices, particularly ICD and CRT, the lack of significance could be influenced by the limited number of patients with such devices. Remarkably other variables such as blood pressure, ischemic etiology and diabetes did not achieve statistical significance in the multivariate analysis and did not improve the model prediction of risk, and were not included in the calculator.

Recently, Ky et al.[23] showed that adding a more complex biomarker panel consisting of high-sensitivity C-reactive protein, myeloperoxidase, B-type natriuretic peptide, soluble fms-like tyrosine kinase receptor-1, troponin I, ST2, creatinine, and uric acid to the Seattle HF Model improves the predictive accuracy for 1-year all-cause death, with a C-statistic up to 0.8. In contrast, both of our clinical and biomarker additive models were less complex but performed similarly. Choosing the panel of biomarkers to deploy in clinical practice will depend on factors such as cost and ease of assay, among others.

### Limitations

Our population was a general HF population treated at a HF unit in a tertiary hospital. Most patients were white and referred from the cardiology department and, thus, relatively young men with HF of ischemic etiology and reduced LVEF. As such, risk prediction is more accurate in these patients. The risk calculator is based on ambulatory patients with chronic HF and may require extensive adjustments when applied to an inpatient population, some of whom have acute decompensated HF. Absence of external validation represents an acknowledged limitation, although we overcame it using 10-fold cross-validation analysis with 1000 bootstrapping as already discussed.

## Conclusion

We developed a new HF risk calculator that incorporates available biomarkers reflecting different pathophysiological pathways and allows quick and easy prediction of death at 1, 2, and 3 years.

## Supporting Information

Figure S1 Graphic with monthly mortality probabilities in the same patient (68 year- old male in NYHA class III, LVEF 30%, sodium 130 mmol/L, eGFR 45 ml/min/m2, hemoglobin 12 g/dl, taking 60 mg of furosemide and on treatment with statins, ACEI and betablockers) according to model without biomarkers (continuous line) and with biomarkers (dashed line). Left panel biomarker values: hs-cTnT 14 ng/L and ST2 40 ng/mL and NTproBNP of 900 ng/L. Righ panel biomarker values: hs-cTnT 70 ng/L, ST2 140 ng/mL and NTproBNP 2500 ng/L.
(TIF)

File S1 Supporting tables.
(DOC)
