# Supplementary figures and images for: Development of a Novel Heart Failure Risk Tool: The Barcelona Bio-Heart Failure Risk Calculator (BCN Bio-HF Calculator)
Source: PLoS One. 2014 Jan 15;9(1):e85466. doi: 10.1371/journal.pone.0085466 (PMC3893213; doi:10.1371/journal.pone.0085466)

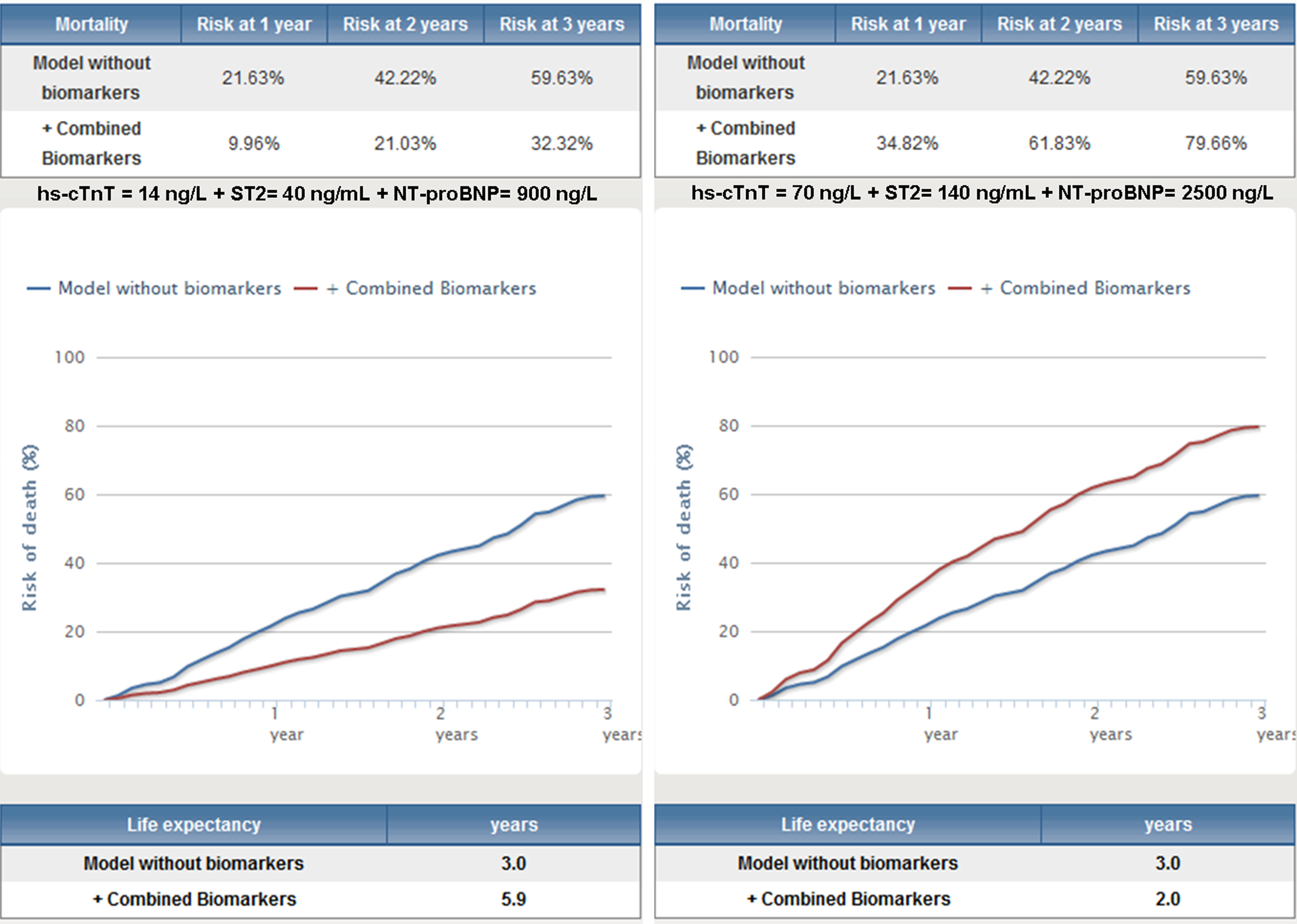

Supplement: Figure S1 — Graphic with monthly mortality probabilities in the same patient (68 year- old male in NYHA class III, LVEF 30%, sodium 130 mmol/L, eGFR 45 ml/min/m2, hemoglobin 12 g/dl, taking 60 mg of furosemide and on treatment with statins, ACEI and betablockers) according to model without biomarkers (continuous line) and with biomarkers (dashed line). Left panel biomarker values: hs-cTnT 14 ng/L and ST2 40 ng/mL and NTproBNP of 900 ng/L. Righ panel biomarker values: hs-cTnT 70 ng/L, ST2 140 ng/mL and NTproBNP 2500 ng/L. (TIF) [file pone.0085466.s001.tif]
