# Supplementary material for: Development of a Novel Heart Failure Risk Tool: The Barcelona Bio-Heart Failure Risk Calculator (BCN Bio-HF Calculator)
Source: PLoS One. 2014 Jan 15;9(1):e85466. doi: 10.1371/journal.pone.0085466 (PMC3893213; doi:10.1371/journal.pone.0085466)
Supplement: File S1 — Supporting tables. (DOC) [file pone.0085466.s002.doc]

**Online supplementary table S1: Beta-coefficients for the different models for 1-, 2-, and 3-year mortality.**

|  | **Model 1** | **Model 2** | **Model 3** | **Model 4** | **Model 5** | **Model 6** | **Model 7** | **Model 8** |
| --- | --- | --- | --- | --- | --- | --- | --- | --- |
| **Age** | 0.04054*** | 0.04037*** | 0.03851*** | 0.03836*** | 0.04045*** | 0.03563*** | 0.03812*** | 0.03659*** |
| **Female sex** | -0.44658** | -0.55148*** | -0.34439** | -0.42336** | -0.47292*** | -0.40813** | -0.26197* | -0.29220* |
| **NYHA** | 0.63749*** | 0.55370*** | 0.56620 | 0.54239*** | 0.51952*** | 0.51716*** | 0.51325*** | 0.51188*** |
| **LVEF** | -0.37702* | --- | --- | --- | --- | --- | --- | --- |
| **Sodium** | -0.06251*** | -0.05737*** | -0.04519** | -0.04592** | -0.04719** | -0.04600** | -0.03523* | -0.03672* |
| **eGFR** | -0.01001* | --- | --- | -0.01088** | --- | --- | --- | --- |
| **Hemoglobin** | -0.12002** | -0.11560** | -0.09213** | -0.12581*** | -0.12687*** | -0.08841* | -0.10332** | -0.09665** |
| **Loop diuretic dose 1**† | 0.22825 | 0.27624 | --- | 0.21859 | 0.27823 | 0.19246 | --- | --- |
| **Loop diuretic dose 2**‡ | 0.56390* | 0.64405** | --- | 0.47031* | 0.54724* | 0.48348* | --- | --- |
| **Statin treatment** | -0.46052*** | -0.37624** | -0.42331*** | -0.38763 | -0.35694** | -0.41989*** | -0.41691*** | -0.39753** |
| **ACEI or ARB treatment** | -0.40481* | --- | -0.31261* | --- | --- | --- | --- | --- |
| **Beta-blocker treatment** | -0.58605*** | -0.58492*** | -0.48259*** | -0.47811*** | -0.56117*** | -0.51714*** | -0.45971** | -0.51180*** |
| **Log(NTproBNP)** | --- | 0.29549*** | --- | --- | 0.24510*** | 0.17359** | **---** | 0.11586* |
| **Log(hs-cTnT)** | --- | --- | 1.48105*** | --- | --- | 1.17561** | 1.46983*** | 1.36975*** |
| **(Log(hs-cTnT))^2^** | --- | --- | -0.14121** | --- | --- | -0.11488* | -0.14633** | -0.14453** |
| **ST2/10** | --- | **---** | **---** | 0.24111*** | 0.22141*** | **---** | 0.24668*** | 0.23017*** |
| **(ST2/10)^2^** | --- | --- | **---** | -0.00842** | -0.00883*** | **---** | -0.00937*** | -0.00898*** |
|  |  |  |  |  |  |  |  |  |
| **Sum product** ¶ | -8.922 | -5.091 | -2.747 | -5.469 | -3.388 | -1.981 | -0.445 | -0.237 |

p-value: * ≤0.05 ** ≤ 0.01 *** ≤ 0.001

ACEI= angiotensin-converting enzyme inhibitor; ARB= angiotensin II receptor blocker; eGFR= estimated glomerular filtration rate; hs-cTnT, high-sensitivity circulating troponin T; LVEF= left ventricular ejection fraction; NT-proBNP= N-terminal pro-brain natriuretic peptide; NYHA= New York Heart Association; ST2= high-sensitivity soluble ST2.

NT-proBNP as log(NTproBNP), hs-cTnT as log(hs-cTnT) and ST2 as ST2/10. The logarithmic functions of NTproBNP and hs-cTnT, the quadratic term of the logarithmic function of hs-cTnT and the quadratic term of ST2/10 were used in the Cox models.

† Loop diuretic dose 1: Furosemide-equivalent dose up to 40 mg/day or Torasemide up to 10 mg/day. Reference category: no loop diuretic treatment.

‡ Loop diuretic dose 2: Furosemide-equivalent dose > 40 mg/day day or Torasemide > 10 mg/day. Reference category: no loop diuretic treatment.

¶ Sum product of coefficients per covariates means

Model 1 (Clinical) = Age, Female, NYHA functional class (as I-II vs III-IV), LVEF (as ≥ vs < 45%), eGFR (estimated glomerular filtration rate), Sodium,

Hemoglobin, Furosemide equivalent doses treatment (as 0, ≤ 40 mg/day and > 40 mg/day), ACEI or ARB treatment, β-blocker treatment, statin treatment.

Model 2= Model 1 + NT-proBNP.

Model 3= Model 1 + hs-cTnT.

Model 4= Model 1 + ST2 .

Model 5= Model 1+ NT-proBNP. + ST2.

Model 6= Model 1+ NT-proBNP + hs-cTnT

Model 7= Model 1 + hs-cTNT + ST2.

Model 8 = Model 1+ NT-proBNP + hs-cTNT + ST2.

**Online Supplementary Table S2: Beta-coefficients for the different Weibull models used to calculate Life Expectancy**

|  | **Model 1** | **Model 2** | **Model 3** | **Model 4** | **Model 5** | **Model 6** | **Model 7** | **Model 8** |
| --- | --- | --- | --- | --- | --- | --- | --- | --- |
| **Intercept** | 1.21230 | 4.21310 | 6.11189 | 3.88479 | 5.57355 | 6.72271 | 7.89796 | 8.04409 |
| **Age** | -0.03255 | -0.03235 | -0.03103 | -0.03080 | -0.03194 | -0.02838 | -0.03019 | -0.02885 |
| **Female sex** | 0.35346 | 0.43129 | 0.27151 | 0.33248 | 0.36486 | 0.31599 | 0.20365 | 0.22476 |
| **NYHA** | -0.50327 | -0.43488 | -0.44838 | -0.42745 | -0.40114 | -0.40355 | -0.39889 | -0.39515 |
| **LVEF** | 0.28803 |  |  |  |  |  |  |  |
| **Sodium** | 0.04919 | 0.04475 | 0.03540 | 0.03634 | 0.03634 | 0.03536 | 0.02718 | 0.02809 |
| **eGFR** | 0.00796 |  |  | 0.00865 |  |  |  |  |
| **Hemoglobin** | 0.09407 | 0.09072 | 0.07259 | 0.09804 | 0.09721 | 0.06897 | 0.07887 | 0.07341 |
| **Loop diuretic dose 1**† | -0.18967 | -0.22488 |  | -0.18215 | -0.22541 | -0.15572 |  |  |
| **Loop diuretic dose 2**‡ | -0.45051 | -0.50759 |  | -0.37536 | -0.42741 | -0.37781 |  |  |
| **Statin treatment** | 0.36816 | 0.30377 | 0.34317 | 0.31289 | 0.28403 | 0.33643 | 0.33238 | 0.31582 |
| **ACEI or ARB treatment** | 0.31485 |  | 0.23985 |  |  |  |  |  |
| **Beta-blocker treatment** | 0.46533 | 0.46174 | 0.38513 | 0.38193 | 0.43710 | 0.40488 | 0.36056 | 0.39653 |
| **Log(NTproBNP)** |  | -0.22807 |  |  | -0.18579 | -0.13107 |  | -0.08560 |
| **Log(hs-cTnT)** |  |  | -1.16717 |  |  | -0.91368 | -1.13491 | -1.05296 |
| **(Log(hs-cTnT))^2^** |  |  | 0.11150 |  |  | 0.08919 | 0.11328 | 0.11121 |
| **ST2/10** |  |  |  | -0.18795 | -0.16907 |  | -0.18767 | -0.17415 |
| **(ST2/10)^2^** |  |  |  | 0.00656 | 0.00674 |  | 0.00713 | 0.00679 |
| $\Gamma(1+\hat{\sigma})$**(*)** | 0.93133 | 0.93033 | 0.93171 | 0.93137 | 0.92747 | 0.92888 | 0.92814 | 0.92690 |

(*) The gamma function parameter obtained from the model

ACEI= angiotensin-converting enzyme inhibitor; ARB= angiotensin II receptor blocker; eGFR= estimated glomerular filtration rate; hs-cTnT, high-sensitivity circulating troponin T; LVEF= left ventricular ejection fraction; NT-proBNP= N-terminal pro-brain natriuretic peptide; NYHA= New York Heart Association; ST2= high-sensitivity soluble ST2.

NT-proBNP as log(NTproBNP), hs-cTnT as log(hs-cTnT) and ST2 as ST2/10. The logarithmic functions of NTproBNP and hs-cTnT, the quadratic term of the logarithmic function of hs-cTnT and the quadratic term of ST2/10 were used in the Cox models.

† Loop diuretic dose 1: Furosemide-equivalent dose up to 40 mg/day or Torasemide up to 10 mg/day

‡ Loop diuretic dose 2: Furosemide-equivalent dose > 40 mg/day day or Torasemide > 10 mg/day

Model 1 (Clinical) = Age, Female, NYHA functional class (as I-II vs III-IV), LVEF (as ≥ vs < 45%), eGFR (estimated glomerular filtration rate), Sodium,

Hemoglobin, Furosemide equivalent doses treatment (as 0, ≤ 40 mg/day and > 40 mg/day), ACEI or ARB treatment, β-blocker treatment,

Statin treatment.

Model 2= Model 1 + NT-proBNP.

Model 3= Model 1 + hs-cTnT.

Model 4= Model 1 + ST2 .

Model 5= Model 1+ NT-proBNP + ST2.

Model 6= Model 1+ NT-proBNP + hs-cTnT.

Model 7= Model 1 + hs-cTNT + ST2.

Model 8 = Model 1 + NT-proBNP + hs-cTNT + ST2.

**Online Supplementary Table S3: Limtis (99^th^ percentile) in the BCN Bio-HF calculator and median values.**

|  | Lower limit | Upper limit | Median |
| --- | --- | --- | --- |
| **Age, years** | 35 | 88 | 70.3 |
| **LVEF, %** | 10 | 71 | 34 |
| **Sodium, mmol/L** | 130 | 147 | 139 |
| **eGFR, ml/min/1.73m2** | 6 | 110 | 42.4 |
| **Hemoglobin, g/dL** | 8.7 | 17.1 | 12.9 |
| **NTproBNP, ng/L** | 21 | 34800 | 1361.5 |
| **hs-cTnT, ng/L** | 3 | 265 | 22.6 |
| **ST2, ng/mL** | 18 | 157 | 38.1 |

eGFR= estimated glomerular filtration rate; hs-cTnT, high-sensitivity circulating troponin T; LVEF= left ventricular ejection fraction; NT-proBNP= N-terminal pro-brain natriuretic peptide; ST2= high-sensitivity soluble ST2.

**Online Supplementary Table S4**: **Limits of life expectancy in the BCN Bio-HF calculator according to age and sex.**

| Age (years) | Men | Women |
| --- | --- | --- |
| ≤63 | >20 | >20 |
| 64 | 19,3 | >20 |
| 65 | 18,5 | >20 |
| 66 | 17, 8 | >20 |
| 67 | 17,0 | >20 |
| 68 | 16,2 | 20,0 |
| 69 | 15,5 | 19,0 |
| 70 | 14,7 | 18,2 |
| 71 | 14,0 | 17,3 |
| 72 | 13,4 | 16,5 |
| 73 | 12,7 | 15,7 |
| 74 | 12,1 | 14,9 |
| 75 | 11,4 | 14,1 |
| 76 | 10,8 | 13,3 |
| 77 | 10,1 | 12,5 |
| 78 | 9,5 | 11,7 |
| 79 | 8,8 | 10,9 |
| 80 | 8,3 | 10,1 |
| 81 | 7,7 | 9,5 |
| 82 | 7,3 | 9,0 |
| 83 | 6,9 | 8,4 |
| 84 | 6,4 | 7,8 |
| 85 | 6,0 | 7,3 |
| 86 | 5,5 | 6,7 |
| 87 | 5,1 | 6,2 |
| 88 | 4,7 | 5,6 |

* The superior limit for life expectancy was set to mean life expectancy in Catalonian population in 2010 ([www.idescat.cat](http://www.idescat.cat/)) or to >20 years if higher.

**Figure legend Online Supplementary Figure S1.**

Graphic with monthly mortality probabilities in the same patient (68 year- old male in NYHA class III, LVEF 30%, sodium 130 mmol/L, eGFR 45 ml/min/m2, hemoglobin 12 g/dl, taking 60 mg of furosemide and on treatment with statins, ACEI and betablockers) according to model without biomarkers (continuous line) and with biomarkers (dashed line). Left panel biomarker values: hs-cTnT 14 ng/L and ST2 40 ng/mL and NTproBNP of 900 ng/L. Righ panel biomarker values: hs-cTnT 70 ng/L, ST2 140 ng/mL and NTproBNP 2500 ng/L.

**APPENDIX**

Using the formula detailed in Methods section and the figures from Online supplementary Table S1 the risk of death of a particular patients may be calculated.

For example, in absence of biomarker information (Model 1), to calculate the probability of dying at 3 years for a 68 year-old male in NYHA class III, LVEF 30%, sodium 130 mmol/L, eGFR 45 ml/min/m2, hemoglobin 12 g/dl, taking 60 mg of furosemide (i.e. Loop diuretic dose 2) and on treatment with statins, ACEI and betablockers:

$\hat{\text{S}}(\text{3years})$= 0.802

$(\overrightarrow{\beta}*\overrightarrow{x_{i}})$= (0.04054 * 68) + (-0.44658 * 0) + (0.63749 * 1) + (-0.37702 * 0) + (-0.06251 * 130) + (-0.01001 * 45) + (-0.12002 * 12) + (0.56390 * 1) + (-0.46052 * 1) + (-0.40481 * 1) + (-0.58605 * 1) = -7.510

Also from Table 2:

$(\overrightarrow{\beta}*\overrightarrow{\bar{x}})$= -8.922

Therefore:

$1-0.802^{\exp^{(-7.510)-(-8.922)}}$= 0.60

i.e. 60% of patients with the above characteristics will died within 3 years.

If biomarker information was available for this patient, e.g. NTproBNP of 900 ng/L, hs-cTnT 14 ng/L and ST2 40 ng/mL, Model 8 would be used and therefore:

$(\overrightarrow{\beta}*\overrightarrow{x_{i}})$= (0.03659 * 68) + (-0.29220 * 0) + (0.51188 * 1) + (-0.03672 * 130) + (-0.09665 * 12) + (-0.39753 * 1) + (-0.51180 * 1) + (0.11586 * log(900)) + (1.36975 * log(14))+ (-0.14453 * log(14)^2)+ (0.23017 * 40/10) + (-0.00898 * (40/10)^2) = 0.331

$(\overrightarrow{\beta}*\overrightarrow{\bar{x}})$= -0.237

Therefore:

$1-0.802^{\exp^{(0.331)-(-0.237)}}$= 0.32

i.e. 32% of patients with the above characteristics will died within 3 years.

Similarly in this patient at 1- and 2-year risk is 22% and 42%, respectively without biomarkers; and 10% and 21%, respectively with the same biomarker levels used to calculate risk at 3 years.

Age, sex, NYHA functional class and treatments are obligatory fields. When LVEF, sodium, haemoglobin or eGFR are missing median values are applied. When any value is out of range (99th percentile), the 99th percentile is applied. Median values and calculation limits (99th percentiles) are displayed in online supplementary Table S3, in File S1. Graphic display of mortality curves were developed using monthly mortality probabilities (online supplementary Fig.S1). The superior limit for life expectancy was set to mean life expectancy in Catalonian population in 2010 ([www.idescat.cat](http://www.idescat.cat/)) or to >20 years if higher, as actual follow-up does not allow to predict so long-term accurately (online supplementary Table S4, in File S1).
